# Herb-Induced Liver Injury in the Berlin Case-Control Surveillance Study

**DOI:** 10.3390/ijms17010114

**Published:** 2016-01-15

**Authors:** Antonios Douros, Elisabeth Bronder, Frank Andersohn, Andreas Klimpel, Reinhold Kreutz, Edeltraut Garbe, Juliane Bolbrinker

**Affiliations:** 1 Institute of Clinical Pharmacology and Toxicology, Charité-Universitätsmedizin Berlin, 10117 Berlin, Germany; elisabeth.bronder@t-online.de (E.B.); andreas.klimpel@t-online.de (A.K.); reinhold.kreutz@charite.de (R.K.); garbe@bips.uni-bremen.de (E.G.); juliane.bolbrinker@charite.de (J.B.); 2 Institute for Social Medicine, Epidemiology and Health Economy, Charité-Universitätsmedizin Berlin, 10117 Berlin, Germany; frank.andersohn@charite.de; 3 Department of Clinical Epidemiology, Leibniz Institute for Prevention Research and Epidemiology—BIPS, 28359 Bremen, Germany

**Keywords:** hepatotoxicity, phytotherapeutics, pharmacovigilance

## Abstract

Herb-induced liver injury (HILI) has recently attracted attention due to increasing reports of hepatotoxicity associated with use of phytotherapeutics. Here, we present data on HILI from the Berlin Case-Control Surveillance Study. The study was initiated in 2000 to investigate the serious toxicity of drugs including herbal medicines. Potential cases of liver injury were ascertained in more than 180 Departments of all 51 Berlin hospitals from October 2002 to December 2011. Drug or herb intake was assessed through a standardized face-to-face interview. Drug or herbal aetiology was assessed based on the updated Council for International Organizations of Medical Sciences scale. In ten of all 198 cases of hepatotoxicity included in the study, herbal aetiology was assessed as probable (once ayurvedic herb) or possible (*Valeriana* five times, *Mentha piperita* once, *Pelargonium sidoides* once, *Hypericum perforatum* once, *Eucalyptus globulus* once). Mean age was 56.4 ± 9.7 years, and the predominant pattern of liver injury was hepatocellular. No cases of acute liver failure or death were observed. This case series corroborates known risks for ayurvedic herbs, supports the suspected association between *Valeriana* use and liver injury, and indicates a hepatotoxic potential for herbs such as *Pelargonium sidoides*, *Hypericum perforatum* or *Mentha piperita* that were rarely associated with liver injury before. However, given that possible causality does not prove clinical significance, further studies in this field are needed.

## 1. Introduction

Intake of herbs has increased over the last years, although data proving efficacy are lacking for most compounds [1]. Several factors may account for this development, including the belief that herbs are safe because they are “natural”, and that they depict a harmless alternative to conventional medicine. Moreover, as they are predominantly non-prescription drugs, they can be easily obtained in drug stores, health stores or via the internet [1]. According to recent data, increased herbal consumption has been accompanied by an increase in cases of herb-induced liver injury (HILI) [2,3,4]. Until today, many different compounds have been linked to liver damage, and for some of them, a high hepatotoxic potential is assumed [5]. However, herbal intake is common even amongst people with existing hepatobiliary disorders [1], probably reflecting the aforementioned attitudes towards herbs in the general population, and the fact that physicians are often unaware of their patients’ use of phytotherapeutics.

Symptoms of HILI can range from mild asymptomatic liver enzyme elevation to more severe conditions such as cirrhosis or acute liver failure (ALF) [5]. A recent prospective cohort study using data from the US Drug-Induced Liver Injury Network (DILIN) showed that hepatic damage from non-bodybuilding herbs and dietary supplements led more often to liver transplantation than conventional drugs (13% *vs.* 3%) [3]. Another US study showed that two-thirds of emergency department visits for single-supplement-related adverse events involved herbal or complementary nutritional products [6]. These findings highlight the significance of HILI and the need to acquire more data on potentially hepatotoxic herbs and characteristics of the respective patients.

Previously, we reported the results of the case-control analysis on drug-induced liver injury from the Berlin Case-Control Surveillance Study FAKOS (Fall-Kontroll-Studie) [7]. The present study aims to contribute further information on the field of HILI. We present a descriptive overview of patients with HILI from the FAKOS study including demographic, clinical, laboratory, and histological features. Moreover, literature on the hepatotoxic potential of the herbs involved is reviewed.

## 2. Results

### 2.1. Case Series

**Figure 1:**
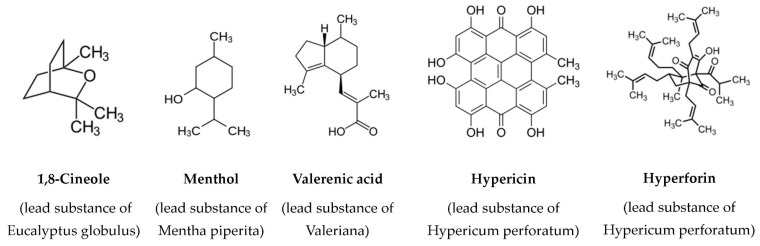
Chemical structures of lead substances of assessed phytotherapeutics.

Of the 198 patients overall with hepatotoxicity initially included into the FAKOS study, ten patients (5.1%) showed a possible or probable herbal aetiology. In seven of these patients, the hepatotoxic reaction occurred in the ambulatory setting requiring hospitalisation, and in the other three patients, it occurred during the hospital stay. Table 1 illustrates demographic, clinical, and laboratory characteristics. Seven patients were female, three patients were male, mean age was 56.4 ± 9.7 years, and the predominant pattern of liver injury according to the ratio R was hepatocellular. Mean alanine aminotransferase (ALT)/upper limit of normal (ULN) ratio was 19.2, mean aspartate aminotransferase (AST)/ULN ratio was 14.3, and mean alkaline phosphatase (ALP)/ULN ratio was 2.3. Five patients depicted hyperbilirubinaemia and coagulopathy was identified in two patients. All serologic tests conducted for viral hepatitis were negative. The most common symptoms were fatigue (seven patients) and jaundice (four patients). No cases of hepatic encephalopathy or ALF were observed, and none of the patients died. Table 2 summarizes the histologic features of the six patients with available liver biopsy results. Five patients showed signs of necrosis either disseminated or predominantly near the central vein (zone III). Portal inflammation was slightly more common than lobular inflammation, and the infiltrates contained mostly lymphocytes, neutrophil or eosinophil granulocytes. Canalicular cholestasis was seen in one patient. Table 3 and Table 4 show the findings based on the updated Council for International Organizations of Medical Sciences (CIOMS) scale: in one case the association between herb intake and hepatotoxicity was assessed as probable (ayurvedic herb) and in nine cases the association was assessed as possible (five times *Valeriana*, once *Mentha piperita*, *Pelargonium sidoides*, *Hypericum perforatum*, and *Eucalyptus globulus*, respectively). Known lead substances of the assessed phytotherapeutics are illustrated in Figure 1. In nine cases, drugs apart from herbs were also suspected as potentially hepatotoxic (Table 3 and Table 4). Regarding the time course of liver enzymes, drug or herb cessation always led to a significant enzymatic decrease.

**Table 1:** Selected demographic, clinical, and laboratory data of the patients with herb-induced liver injury.

	1	2	3	4	5	6	7	8	9	10
Suspected herb	Ayurvedic herb	*Hypericum perforatum*	*Valeriana*	*Valeriana*	*Mentha piperita*	*Pelargonium sidoides*	*Eucalyptus globulus*	*Valeriana*	*Valeriana*	*Valeriana*
Sex	Female	Female	Female	Female	Male	Female	Male	Male	Female	Female
Age (years)	60	65	57	70	46	52	50	46	71	47
Grading of liver injury	Hepatocellular	Mixed	Cholestatic	Hepatocellular	Hepatocellular	Hepatocellular	Hepatocellular	Hepatocellular ^1^	Cholestatic	Non classifiable
Laboratory testing										
ALT/ULN	34.9	19.2	7.5	3.5	73.6	34.7	6.1	3.5	4.1	4.6
AST/ULN	49.2	12.8	4.7	4.6	44.6	15.2	3.1	3.3	3.4	2.5
ALP/ULN	1.0	5.2	5.8	0.6	2.0	1.6	0.4	0.8	3.1	Missing
Bilirubin total (mg/dL)	35.3	2.0	7.1	7.8	3.8	1.0	0.5	0.5	0.5	Missing
Coagulopathy ^2^	Yes (INR: 1.6)	No	No	Yes (INR: 1.9)	No	No	No	No	No	No
Serology testing										
Hepatitis A virus	Negative	Negative	Negative	Negative	Negative	Negative	Missing	Negative	Negative	Negative
Hepatitis B virus	Negative	Negative	Negative	Negative	Negative	Negative	Negative	Negative	Negative	Negative
Hepatitis C virus	Negative	Negative	Negative	Negative	Negative	Negative	Negative	Negative	Missing	Negative
Cytomegalovirus	Negative	Negative	Missing	Missing	Missing	Missing	Negative	Missing	Missing	Missing
Epstein-Barr virus	Negative	Negative	Missing	Missing	Missing	Missing	Negative	Missing	Missing	Missing
Hepatitis E virus	Negative	Missing	Missing	Missing	Missing	Missing	Missing	Missing	Missing	Missing
Herpes simplex virus	Negative	Missing	Missing	Missing	Missing	Missing	Negative	Missing	Missing	Missing
Varicella zoster virus	Negative	Missing	Missing	Missing	Missing	Missing	Negative	Missing	Missing	Missing
Autoimmune antibodies testing	ANA 1:320	Negative	ANA 1:160	Negative	Negative	Negative	Missing	Negative	Negative	Missing
Abdominal sonography	Conducted	Conducted	Conducted	Conducted	Conducted	Conducted	Conducted	Conducted	Conducted	Conducted
Symptoms										
Fatigue	Yes	Yes	Yes	Yes	Yes	Yes	No	Yes	No	No
Jaundice	Yes	No	Yes	Yes	Yes	No	No	No	No	No
Acholic faeces/dark urine	Yes	No	Yes	No	Yes	No	No	No	No	No
Abdominal pain	No	Yes	Yes	No	No	No	No	No	No	No
Signs of hypersensitivity ^3^	Yes	No	No	No	Yes	No	No	No	No	No
Hepatic encephalopathy	No	No	No	No	No	No	No	No	No	No
Acute liver failure ^4^	No	No	No	No	No	No	No	No	No	No
Death	No	No	No	No	No	No	No	No	No	No

^1^ As ALP remained normal, the pattern of liver injury was classified as hepatocellular although the ratio ALT/ULN/ALP/ULN was lower than 5 (4.4); ^2^ INR > 1.2; ^3^ Fever, rash, lymphadenopathy, arthralgia, myalgia and/or eosinophilia; ^4^ Severe coagulopathy (INR > 1.5) and hepatic encephalopathy. ALT = alanine aminotransferase; ULN = upper limit of normal; AST = aspartate aminotransferase; ALP = alkaline phosphatase; ANA = antinuclear antibodies; INR = International Normalized Ratio.

**Table 2:** Histologic features of the patients with herb-induced liver injury.

Histologic Features	1	2	3	4	8	9
Suspected herb	Ayurvedic herb	*Hypericum perforatum*	*Valeriana*	*Valeriana*	*Valeriana*	*Valeriana*
Necrosis	+++	+	+	+	+	−
Necrosis localisation	Zone III	Disseminated	Disseminated	Zone III	Disseminated	Not applicable
Lobular inflammation	+	+	+	+	+	−
Portal inflammation	++	++	++	+	−	+
Plasma cells	++	−	+	?	−	−
Eosinophils	+	+	−	?	+	−
Neutrophils	++	−	+	?	+	−
Lymphocytes	−	++	++	?	−	+
Canalicular cholestasis	−	−	++	−	−	−

? = no data available.

**Table 3:** Data on drug or herb intake of the patients with herb-induced liver injury and hepatocellular patterns.

	1	4	5	6	7	8	10 ^1^
Causality assessment ^2^							
probable	**Ayurvedic herb ^3^ (6)**	-	ASA/Vitamin C (6)	-	Clarithromycin (6)	-	-
possible	Metoprolol (4)	Metformin (5)	***Mentha piperita* (3)**	***PS* (4)**	***Eucalyptus globulus* (3)**	Venlafaxine (4)	***Valeriana* (4)**
	-	Paroxetine (5)	-	-	Multivitamins (3)	***Valeriana* (3)**	Citalopram (3)
	-	***Valeriana* (4)**	-	-	-	-	-
	-	Multivitamins (3)	-	-	-	-	-
Time to onset from the beginning of drug/herb							
5–90 days	**Ayurvedic herb**	-	-	-	All	-	All
<5 or >90 days	Metoprolol	All	All	-	-	***Valeriana***	-
OR: Time to onset from cessation of drug/herb ≤15 days (except for slowly metabolized chemicals: >15 days)	-	-	-	***PS***	-	Venlafaxine	-
Course of ALT after cessation of the drug/herb (percentage difference between ALT peak and N)	-	-	-	-	-	-	-
Decrease ≥50% within 8 days	-	-	All	-	All	-	-
Decrease ≥50% within 30 days	-	All	-	-	-	All	All
No information	All	-	-	***PS***	-	-	-
Decrease ≥50% after the 30th day							
Decrease <50% after the 30th day or recurrent increase	-	-	-	-	-	-	-
ALT normalization after cessation of drug/herb	Not known	Not known	Not known	Not known	Yes (30 days)	Yes (10 days)	Not known

^1^ The pattern of liver injury could not be assessed as no ALP values were available and no liver biopsy was conducted; ^2^ Based on the updated CIOMS score as proposed by Teschke *et al.* [4]; ^3^ Tea containing liquorice, ginger, cardamom, and cinnamon. Herbs are written in bold. Numbers in brackets depict the points in CIOMS score for each compound. ASA = acetyl salicylic acid; PS = *Pelargonium sidoides*; ALT = alanine aminotransferase; N = enzyme value at a certain time point; CIOMS = Council for International Organizations of Medical Sciences.

**Table 4:** Data on drug or herb intake of the patients with herbal-induced liver injury and cholestatic or mixed patterns.

	2	3	9
Causality assessment ^1^	-	-	-
probable	-	-	-
possible	***Hypericum perforatum* (5)**	Carbimazole (5)	Enoxaparin (4)
	Promethazine (5)	Phenprocoumon (5)	Metamizole (3)
	-	***Valeriana* (3)**	***Valeriana* (3)**
Time to onset from the beginning of drug/herb			
5–90 days	***Hypericum perforatum***	Carbimazole, Phenprocoumon	All
<5 or >90 days	Promethazine	***Valeriana***	-
OR: Time to onset from cessation of drug/herb ≤15 days (except for slowly metabolised chemicals: >15 days)			
Course of ALP after cessation of the drug/herb (percentage difference between ALP peak and N)	-	-	-
Decrease ≥50% within 180 days	All	-	-
Decrease <50% within 180 days	-	All	All
No information, persistence, increase, or continued drug/herb use	-	-	-
ALP normalisation after cessation of the drug/herb	Not known	Not known	Not known

^1^ Based on the updated CIOMS score as proposed by Teschke *et al.* [4]. Herbs are written in bold. Numbers in brackets depict the points in CIOMS score for each compound. ALP = alkaline phosphatase; N = enzyme value at a certain time point; CIOMS = Council for International Organizations of Medical Sciences.

### 2.2. A Case with Probable Herbal Causality

Patient 1 used an ayurvedic tea containing liquorice, ginger, cardamom, and cinnamon several times. Within two weeks, she developed jaundice, fatigue, abdominal pain, and an exanthema and was hospitalised. Except for the aforementioned herb, the patient received metoprolol 50 mg daily for the past 14 years due to arterial hypertension with no complications. Laboratory analysis showed strongly increased transaminases and bilirubin, coagulopathy, as well as slightly increased antinuclear antibodies (Table 1). Liver biopsy was compatible with the enzymatic pattern of hepatic damage revealing hepatocellular necrosis as the main characteristic (Table 2). Viral or biliary aetiologies were excluded, alcohol intake was denied. The herb and the beta blocker were stopped and a treatment with corticosteroids was initiated. The patient showed clinical improvement and a decrease of the transaminases in the next three weeks (AST: about 80% reduction; ALT: about 20% reduction) and was thereafter discharged from the hospital. The causality of the metoprolol was classified as “possible” due to its less indicative time course.

### 2.3. A Case Associated with the Use of Pelargonium sidoides

Patient 6 received *Pelargonium sidoides* as drops due to a common cold for five days. A few days later she developed fatigue, and two weeks after herb cessation, ambulatory control of liver enzymes showed strongly increased transaminases. In hospital, a limited serologic analysis (including only hepatitis A virus, hepatitis B virus and hepatitis C virus) did not reveal any signs of viral hepatitis and abdominal imaging excluded a biliary aetiology (Table 1). Clinical improvement and fast decrease of both transaminases were followed by patient’s discharge. Although no other medications were compatible with the time course of the illustrated symptoms, as dechallenge assessment was performed long after stopping herb use, only a possible aetiology can be attributed to *Pelargonium sidoides* (Table 3).

## 3. Discussion

This series provides data from ten well phenotyped hepatotoxicity cases with probable or possible herbal aetiology. Since, in FAKOS, the overall number of hepatotoxicity cases was 198, liver damage associated with herbs seems to be rare in our data (circa 5%). The US DILIN study showed that 15.5% of hepatotoxicity cases were associated with herb intake [3], suggesting a greater relevance for HILI in the US than in Germany where FAKOS was conducted. However, HILI cases in DILIN cumulated drastically during study period, beginning from 7% of the overall cases in 2004 and reaching 20% in 2014. As case recruitment in FAKOS spanned from 2002 to 2011, we assume that not only country-specific but also time-specific factors may account for this difference. The most common pattern of liver injury in our series was hepatocellular (60%). This is in accordance with the DILIN publication, in which the respective number was 71% [3]. Sex distribution in cases is also comparable, as in both studies mainly female patients developed liver damage associated with herb intake (70% in FAKOS *vs.* 65% in DILIN). As consumption of phytotherapeutics is generally more widespread among women [8], the observed female “predominance” regarding HILI could derive from the higher exposure.

In our case series, the causality of one herb was assessed as probable (ayurvedic herb in patient 1). Regarding ayurvedic treatment, several reports on hepatotoxicity were published in the last years, with most of them referring to *Centella asiatica*, an herb used for various psychiatric, cardiologic or dermatologic indications [9,10,11]. Patient 1 received a tea containing several ingredients, making precise assumptions regarding the hepatotoxic potential of the specific components challenging. Although metoprolol-induced hepatotoxicity cannot be ruled out (Table 3), this case further supports a hepatotoxic potential for ayurvedic herbs.

Concerning *Pelargonium sidoides*, the Drug Commission of the German Medical Association reported several cases of hepatotoxicity associated with its use in 2011 [12]. However, a re-evaluation based on the updated CIOMS score revealed no highly probable or probable causalities [13]. In our case, the causality was assessed as possible. Other drugs were excluded as possible aetiologies, but serologic testing was limited and dechallenge was assessed long after herb use cessation. On the regulatory level, the European Medicines Agency stated in 2013 that “hepatotoxicity and hepatitis cases were reported in association with administration of *Pelargonium sidoides*” and that “in case signs of hepatotoxicity occur, its administration should be stopped immediately and a medical doctor should be consulted” [14]. Accordingly, the Federal Institute for Drugs and Medical Devices in Germany imposed respective changes in labelling [15]. Altogether, a hepatotoxic potential of *Pelargonium sidoides* cannot be excluded, and considering the low quality of data regarding its efficacy in upper respiratory tract infections [16], it should be used cautiously.

Liver damage associated with *Valeriana* has been reported in the past [17,18,19,20], although none of the ingredients of its extracts (e.g., valerenic acid, Figure 1) are known to be specifically hepatotoxic [21]. In our series, all five cases assessed showed a possible causality. The symptoms were mild, the pattern of liver injury varied, and the enzymatic changes were moderate. In our previously published case-control study *Valeriana* showed an increased risk, although statistical significance was scarcely missed (odds ratio 5.3, 95% confidence interval 0.98–27.3) [7]. Regarding the three other herbs assessed as potentially hepatotoxic (*Hypericum perforatum*, *Mentha piperita*, *Eucalyptus globulus*), existing literature is limited. *Hypericum perforatum* was linked to liver damage in two case reports, with the authors suggesting drug–drug interactions as a potential triggering mechanism [22,23]. No data exist regarding the hepatotoxic potential of its two main compounds hyperforin and hypericin (Figure 1). In our case the hepatotoxic causality remains unclear, as the co-administered promethazine also showed a possible causality and jaundice is listed as a known adverse event in its Summary of Product Characteristics [24]. A risk quantification conducted in our case-control study could also not sufficiently clarify the hepatotoxic potential of *Hypericum perforatum* due to the low number of exposed cases and controls (odds ratio 2.9, 95% confidence interval 0.2–35.3) [7]. To our knowledge, no published cases of liver damage linked to the use of *Mentha piperita* exist. An *in vitro* study showing a hepatotoxic potential for high-dose *Mentha piperita* proposed a mechanism involving not its main compound menthol (Figure 1), but pulegone, a monoterpene found in low concentrations in oil extracts which can cause acute hepatic necrosis in *in vivo* experimental models [25]. This compound could also be responsible for the cases of liver injury associated with other members of the same genus such as *Mentha pulegium* [26,27,28,29] or *Mentha haplocalyx Brig* [30]. Finally, despite the existence of preclinical data implicating a hepatotoxic potential for the main compound of *Eucalyptus globulus* 1,8-cineole (Figure 1), involving central venous congestion, granular degeneration, vacuolar degeneration and hepatic necrosis [31,32], there are no cases of liver damage in association with its use.

Some limitations of this study need to be addressed. Firstly, herbs gathered equal or more points than “conventional” drugs in the updated CIOMS score only in four of the ten reported cases. Therefore, the possibility of a non-herb drug aetiology cannot be excluded in most patients. Secondly, since HILI is a diagnosis of exclusion and relatively common hepatitis causes such as infections by cytomegalovirus, Epstein-Barr virus, hepatitis E virus (HEV), herpes simplex virus or varicella zoster virus were rarely excluded in our patients, an infectious aetiology cannot be ruled out for every case. This is particularly important for HEV, since recent publications suggest that although accounting for some cases of suspected drug-induced liver injury, serologic testing excluding a respective infection is not regularly performed [33,34]. Finally, case series are not suitable for risk quantification due to the missing denominator, and definite conclusions regarding clinical characteristics or outcomes cannot be provided. However, similarly to spontaneous adverse drug reports, they can serve as a tool to produce risk signals and raise awareness of potential safety problems [35].

Altogether, our data illustrate a further contribution to the field of HILI. They corroborate known risks for *Valeriana* and ayurvedic herbs and implicate a hepatotoxic potential for *Pelargonium sidoides*, *Hypericum perforatum*, and other compounds only seldom or never linked to liver damage thus far. They also demonstrate the challenges regarding causality assessment of HILI cases, particularly when it comes to the exclusion of alternative diagnoses since respective data are often missing. Therefore, further thoroughly conducted epidemiological studies quantifying hepatotoxic risks for various herbs remain indispensable.

## 4. Patients and Methods

### 4.1. Case Identification and Recruitment

The hospital based case-control surveillance study FAKOS was initiated in 2000 in order to study serious toxicity of drugs including hepatotoxicity [7,36,37]. Potential cases of liver injury were ascertained in more than 180 Departments of Internal Medicine, Neurology, Psychiatry, and Anaesthesiology of all 51 Berlin hospitals from October 2002 until December 2011. The physicians in these departments were contacted regularly in two- to three-week intervals to identify potential cases, and between these intervals cases were actively reported to the study center. Eligible patients were contacted by a trained staff member of FAKOS in order to obtain the patients’ written informed consent. Moreover, a standardized face-to-face interview was conducted, ascertaining information on all previous intakes of drugs or herbs, on co-morbidities, demographic data, and other possible risk factors such as chemicals or solvents. Intake of drugs or herbs was also ascertained by reviewing medical charts. Berlin, with 2.8 million inhabitants as an adult source population, comprised the study region. The Ethics Committee of the Charité-Universitätsmedizin Berlin approved the study (Date: 17 July 2000; Case number: 1369/2000).

### 4.2. Case Definition, Validation and Characterization

Patients with a minimum age of 18 years and a new diagnosis of hepatotoxicity within the last six months were included in the study. Inclusion laboratory criteria were an elevation of ALT or AST threefold above ULN or an elevation of total bilirubin higher than 2 mg/dL. Excluded were patients with underlying liver disease (including, amongst others, acute or chronic infectious hepatitis, alcoholic fatty liver disease, autoimmune hepatitis, liver tumors or hepatic metastases, extrahepatic bile duct obstruction, ischaemic hepatitis or congestive hepatopathy). Hepatotoxicity was validated as certain, probable, possible or unlikely based on information about laboratory, imaging or histological tests and clinical information including exclusion criteria provided on a standardised form by the treating physician to the study center. Only patients with certain or probable hepatotoxicity were further considered. Grading as hepatocellular, cholestatic or mixed type was based on the determination of the ratio R of the relative rise of ALT to the relative rise of ALP [38]. When ALT or ALP was lacking and a biopsy had not been conducted, hepatotoxicity was graded as unclassifiable. Coagulopathy was defined as an International Normalized Ratio (INR) higher than 1.2, hyperbilirubinaemia as total bilirubin higher than 1.1 mg/dL, and ALF as severe coagulopathy (INR > 1.5) combined with hepatic encephalopathy.

### 4.3. Standardized Assessment of Drug or Herbal Causality in Individual Cases

A possible drug or herbal aetiology was assessed for each case by applying the updated CIOMS scale [39]. Two different CIOMS subscales exist, with the first being for the hepatocellular type of liver injury and the second for the cholestatic or mixed type. The updated CIOMS scale takes into account (i) information on the time interval from the beginning of drug or herbal intake until the onset of symptoms or laboratory abnormalities; (ii) the time course of dechallenge; (iii) the existence of risk factors such as age ≥55 years or high alcohol consumption; (iv) concomitant medications; (v) the exclusion of alternative causes such as viral hepatitis, hepatobiliary diseases, alcoholism, ischaemia or complications of underlying diseases; (vi) previous information on hepatotoxicity of the drug or herb; and (vii) a response to unintentional readministration. According to this scale, drug or herbal causality can be “highly probable” (score ≥ 9), “probable” (score 6–8), “possible” (score 3–5), “unlikely” (1–2) or “excluded” (score ≤ 0).
